# *“It is about how the net looks”:* a qualitative study of perceptions and practices related to mosquito net care and repair in two districts in eastern Uganda

**DOI:** 10.1186/1475-2875-13-504

**Published:** 2014-12-17

**Authors:** Leah Scandurra, Angela Acosta, Hannah Koenker, Daniel Musoke Kibuuka, Steven Harvey

**Affiliations:** Johns Hopkins University Center for Communication Programs, Baltimore, MD USA; Service For Generations (SFG) International, Kampala, Uganda; Department of International Health, Johns Hopkins Bloomberg School of Public Health, Baltimore, MD USA

**Keywords:** Malaria, Qualitative research, Direct observation, BCC, ITN, LLIN, Bed nets, Uganda, Net care and repair

## Abstract

**Background:**

Prolonging net durability has important implications for reducing both malaria transmission and the frequency of net replacement. Protective behaviour, such as net care and repair, offers promise for improving net integrity and durability. Given the potential cost-savings and public health benefit associated with extending the useful life of long-lasting insecticidal nets (LLINs), prevention and mitigation of damage will become ever more critical to ensuring adequate net coverage at the population level.

**Methods:**

A qualitative assessment was conducted in two districts in central eastern Uganda in September 2013. Data on household net care and repair behaviour, attitudes and practices were collected from 30 respondents through in-depth interviews (IDIs), observations, photos, and video to gather an in-depth understanding of these behaviours.

**Results:**

Net damage was common and the most cited causes were children and rodents. Responses revealed strong social norms about net cleanliness and aesthetics, and strong expectations that others should care for and repair their own nets. Respondents were receptive and able to repair nets, though longer-term repair methods, such as sewing and patching, were not as commonly reported or observed. Self-reported behaviour was not always consistent with observed or demonstrated behaviour, revealing potential misconceptions and the need for clear and consistent net care and repair messaging.

**Conclusions:**

Respondents considered both aesthetics and malaria protection important when deciding whether, when, and how to care for and repair nets. BCC should continue to emphasize the importance of maintaining net integrity for malaria prevention purposes as well as for maintaining aesthetic appeal. Additional research is needed, particularly surrounding washing, drying, daily storage routines, and gender roles in care and repair, in order to understand the complexity of these behaviours, and refine existing or develop new behaviour change communication (BCC) messages for net care and repair.

## Background

Malaria prevention with long-lasting insecticidal mosquito nets (LLIN) has seen a tremendous scale-up in sub-Saharan Africa in recent years. As many countries are approaching universal coverage targets, it is important to plan how high coverage levels can be maintained. According to World Health Organization Pesticide Evaluation Scheme (WHOPES) specifications, LLINs are expected to confer protection against mosquitoes for at least 20 washes under laboratory conditions and three years of use under field conditions [1]. Recent studies have shown that LLIN durability can vary depending on product-specific or environmental issues such as region, socio-economic status, and climatic conditions [2–6].

Prolonging LLIN durability could reduce the frequency of net replacement, potentially resulting in significant cost-savings in procurement and distribution. Keeping nets in good condition is essential to ensuring household use: studies in Ethiopia, Ghana, Kenya, and Senegal found that nets were used regularly when new, but increasingly withdrawn from use as their physical condition deteriorated [7–10].

Household practices, such as frequency and method of washing and drying, affect net longevity [2, 6, 11, 12]: beating nets on rocks or hanging them in the sun may deplete their insecticide and cause fabric to wear prematurely, making nets less protective against mosquitoes [9, 12, 13]. Alternatively, care behaviour, such as proper washing frequency, careful handling, and tying up nets when not in use may mitigate wear and tear [5, 12, 14]. Protective care behaviour may also improve owner perceptions of net effectiveness and foster more consistent use [8, 9, 15]. Given the potential significant cost-savings and public health benefit associated with extending the useful life of a net, preventing and mitigating damage will become ever more critical to maintaining adequate LLIN coverage [16].

**Figure 1:**
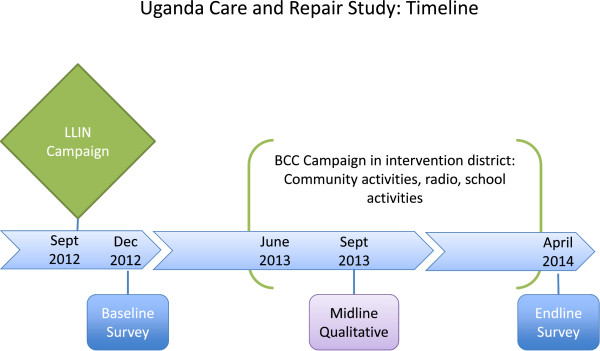
**Net care and repair in Uganda study timeline.**

From 2012–2014, the Uganda National Malaria Control Programme distributed over 20 million LLINs through universal coverage campaigns nationwide. Although net care and repair has been promoted in previous campaigns, specific information about the motivators, barriers, attitudes, and household roles influencing net care and repair has not been studied in Uganda.This paper presents findings from a qualitative assessment, conducted in two districts in eastern Uganda: Serere and Kaliro, two months after the launch of a multi-platform behaviour change communication (BCC) campaign on net care and repair (see Figure 1 for graphic overview of the larger study design). The aim of this assessment was to take an in-depth look at net care and repair behaviour using a variety of methods. Results of quantitative baseline and endline surveys assessing the impact of the BCC campaign will be presented in a forthcoming manuscript.

## Methods

### Study area and population

The two study districts are geographically, culturally, and socio-economically similar, yet speak different languages. As of 2013, the population of Kaliro was 216,500; the population in Serere was 294,100 [17]. Both districts received LLINs from the universal coverage campaign in September 2012. A mix of brands were distributed: PermaNet® brand nets represented 93% of all nets distributed in the intervention district. Olyset®Net brand nets were distributed to 100% of the control district.

### Sample size and study design

Thirty in-depth interviews (IDIs) were conducted, 15 from each district. Participants were purposively selected to maximize variation in household characteristics and experiences of respondents. Eligibility criteria included individuals who were at least 18 years of age, owned at least one net hanging over a sleeping space, and were at least partially responsible for net maintenance and care in the household. Participants included ten men, ten women with or without children of any age, and ten women with children under-five (see Table 1).

**Table 1:** **Study design**

Data collection method	Participant profile	N	N	N
		Kaliro	Serere	Total (combined)
Household visit IDI	Male net user	5	5	10
Household visit IDI	Female net user	5	5	10
Household visit IDI	Female net user w/child under 5	5	5	10
Photo exercises	All participants	15	15	30
Photographs	All participants	15	15	30
Video	All participants	15	15	30
Observations	All participants	15	15	30

IDIs covered aspects of daily net use, net care and repair behaviour, and recall of the BCC campaign. The net hanging over the sleeping space used by the respondent and/or others in the household the night before the interview was the primary net asked about, observed and used during demonstrations. Researchers also noted the condition of other nets they observed in the home during the household interviews, however the net hanging over the sleeping space used by respondents was the primary net for which responses were generated.

To assess the care and repair behaviour of interest, respondents were asked to describe, and then demonstrate in simulated fashion, how they washed, dried, repaired, stored, hung and took down their net and how they prepared it for nightly use. Since actual washing would have taken several hours, field workers asked participants to demonstrate washing without using water or soap.

Audio recordings, observation notes and photographs were collected throughout the interview. Video footage was collected primarily during the simulated demonstrations to capture the entire process of the behaviour.

To assess attitudes regarding damage to nets, respondents were shown five pictures of nets in random order: (1) a clean net with no holes; (2) a somewhat dirty net with no holes; (3) a very dirty net with no holes; (4) a clean net with a few holes; and, (5) clean net with many holes. Participants were asked to describe what they thought of each net, whether the net would be good to sleep in, and if they would use it in their household. To assess attitudes regarding repair methods, respondents were presented with photographs of nets repaired by (1) tying a knot; (2) stitching or sewing; and, (3) patching, then asked for their opinions on each type of repair.

### Data handling and analysis

Audio recordings and textual notes were transcribed and translated verbatim into English immediately after each interview. Transcripts, photographs and videos were coded in Atlas.ti, a qualitative data analysis programme, using both predetermined codes based on research questions and codes that emerged from the data. Once coded, quotes were indexed and analysed for the most salient themes. Researchers reviewed photographs and videos and recorded observations in a spreadsheet, allowing them to compare the reported behaviour from the transcripts with the observed behaviour. Researchers also entered the results from the photograph exercises into spreadsheets to allow them to compare and quantify responses. Researchers convened several times throughout the analysis process to discuss emerging themes and align their interpretations.

### Ethical considerations

Ethical approval was secured from the Johns Hopkins University Bloomberg School of Public Health Institutional Review Board in Baltimore, Maryland, USA (IRB #4534) as well as the Joint Clinical Research Center and the Uganda National Council of Science and Technology in Kampala, Uganda. Participants provided oral consent for all research activities and received two bars of laundry soap after the interview as an incentive for participating in the study.

## Results

### Demographic characteristics

Study participants included five men and ten women from each district ranging in age from 20 to 79 years (median = 38). Participating households from the two districts had similar sources of income and similar numbers of children under five, household members, and nets per household (see Table 2).

**Table 2:** **Demographic characteristics: by district and combined**

Characteristics	Kaliro N (%)	Serere N (%)	Total (combined) N (%)
**Sex**			
Male	5 (17)	5 (17)	10 (34)
Female	10 (33)	10 (33)	20 (67)
**Age**			
≤19	0 (0)	0 (0)	0 (0)
20-29	6 (20)	1 (3)	7 (23)
30-39	3 (10)	5 (17)	8 (27)
40-49	1 (3)	4 (13)	5 (17)
50+	4 (13)	5 (17)	9 (30)
Don’t know	1 (3)	0 (0)	1 (0)
Mean age totals	40	45	42.5
**Household Composition**			
Mean number of children under 5	1.6	1.6	1.6
Mean household size	7.6	6.8	7.2
**Household Characteristics**			
**Roofing**			
Grass/papyrus/banana/thatch	5 (33)	4 (27)	9 (30)
Zinc/iron/tile	10 (67)	11 (73)	21 (70)
**Walls**			
Grass/mud	5 (33)	4 (27)	9 (30)
Plaster/brick	10 (67)	11 (73)	21 (70)
**Floor**			
Earth/sand/clay	4 (47)	9 (60)	13 (43)
Wood/bamboo/palm	3 (10)	0 (0)	3 (10)
Vinyl/tile/cement	8 (53)	6 (40)	14 (47)
**Income**			
Subsistence agriculture	7 (23)	6 (20)	13 (43)
Commercial agriculture	2 (7)	1 (3)	3 (10)
Business owner	6 (20)	4 (13)	10 (33)
Other	0 (0)	4 (13)	4 (13)
**Education**			
Primary	9 (30)	6 (20)	15 (50)
Secondary	2 (7)	2 (7)	4 (13)
Higher	2 (7)	4 (13)	6 (20)
None	2 (7)	3 (10)	5 (17)
**Net Ownership**			
Mean number of nets in household	2.1	3.3	2.7

### Causes of damage

The most commonly cited agents of holes were children and rats. Children were reported to handle nets roughly, tugging at them or poking them with pencils, sticks, knives, or other sharp objects. Participants stated that children’s nets were more likely to have holes than those used by adults, and their nets became dirty more quickly, requiring more frequent washing. Participants said that rats were attracted to nets when children touched them with food on their hands, or when a net touched the ground due to the way it was hung or stored. Other pests such as termites, cockroaches and scorpions were also cited as causes of holes, although less frequently and in less detail. As one respondent explained,

*…when I had just got this net, one of my little children used a knife on [the net]*

*which brought holes in it. She wanted to see if the net was strong. What causes holes the most? I think it is the cockroaches or rats… anyway the rats are the worst because they are very heavy even just falling on the bed [they] can cause tears on the net. Also they have no food so they can eat the net.* (45 year old male, control district)

Other causes had to do with day-to-day household activities. Participants noted that how users handled their nets affected how quickly nets developed holes. Some owners were said to be more likely to tuck nets roughly under mattresses, while others were more likely to handle nets carefully when preparing for bed. Characteristics of sleeping spaces, such as mats, beds or mattresses with rough edges and springs or loose nails were also mentioned as causes of holes. Several respondents mentioned that washing nets frequently caused them to develop holes and that older nets were more likely to tear during washing.

### Attitudes about torn or unclean nets

#### Usability

Presented with photographs of nets with different combinations of holes and dirt, most participants stated that the clean net with no holes was good to sleep in. Most said that the net with a few small holes was still useful; some reported using similar nets in their household. Several respondents mentioned that small holes did not need to be repaired if that portion of the net could be tucked under a mattress. Most participants commented that the dirty and very dirty nets could be used, but only after washing. Participants overwhelmingly rejected the clean net with many holes explaining that the holes were too large or too numerous and could not be repaired, rendering the net unusable. As one respondent explained,

*When a net has big holes that can’t be repaired everywhere, then I can’t use it any longer because it won’t stop mosquitoes from entering so it will not be serving any purpose. This happens when the net is too old, even when you stitch, it just tears more the next day.* (74 year old female, control district)

#### Social norms

Asked what they think when they see a torn net hanging over someone’s bed or sleeping space, the vast majority of participants responded that they expected the owner to either repair or replace the net. If a person did not repair or replace a damaged net, participants described them as ‘irresponsible’ or ‘lazy’, often adding that such owners did not value their health. Many noted that mosquitoes could enter a hole in the net, placing families at risk of malaria.

*Maybe they have no money to buy a new net, but the owner is also lazy. There are simple ways to repair a net, like sewing with needle and thread. So even if you are found with patches, it is evident that you care… The person with a net with holes does not know about the relevance of the net.* (24 year old female, intervention district)

Many informants noted that a good-looking net reflected well on the owner and on the household. As one respondent explained:

*An attractive net is important … because the bed looks good when laid. Even when a visitor enters your bedroom, they can smile and appreciate the order in your room. Allowing a neighbor to see you using a torn or dirty net would be embarrassing*. (34 year old female, control district)

Many participants linked dirty nets with shame and gossip, saying that people would hide dirty nets from visitors and that dirty nets would be laughed at. When asked what people think when they see a dirty net hanging over someone’s bed or sleeping space, none of the participants expected the net to be discarded, but the vast majority expected it to be washed. In one participant’s words:

*Whenever a net is dirty, a person can tell that all aspects of your life are very dirty. I love clean things and I expect even my neighbors to love them. I would think that person is nonsensical and they don’t understand because how can you be defeated to wash a net which doesn’t even take more than 5 minutes to wash up? You wash as if you are doing your laundry, you just touch gently and it gets clean. Why would anyone be defeated?* (45 year old male, control district)

#### Care/maintenance

When asked to describe care practices, respondents cited careful handling, hanging, washing, and keeping nets out of children’s reach by putting them away or tying them up. The majority mentioned washing their nets and putting them away as part of household routines. As one respondent noted,

*[Putting a net away] is like eating food daily, you have to cook, to bathe, so I categorize that together with nets.* (66 year old female, intervention district).

Although only a few respondents mentioned any barriers to care, several respondents mentioned that livelihood responsibilities sometimes interfered with net care routines. As one respondent explained:

*…when it reaches morning and I don’t have any distractions I roll it properly then throw it over the top. But if I wake up in the morning and I have so many distractions, I leave it that way and rush off to the garden. When I return from the [fields] … I fix this place*. (27 year old female, control district)

Respondents demonstrated hanging and taking down their nets and some videos revealed potentially damaging practices, such as hanging nets with nails, overstretching them to fit over beds that were too large, or tucking them into metal or wooden bedframes or under straw mats on the floor.

Most respondents described and demonstrated washing in a way that was unlikely to cause damage. During simulated washing, informants demonstrated washing their nets by stirring or kneading gently in a bucket of water with soap or detergent, rather than scrubbing or beating on the rocks. In washing videos, no respondents were captured washing their nets in a damaging manner. This suggests that washing methods may be a less important source of holes and wear in this context than washing frequency. Although most participants reported washing their nets once every few weeks, a few said they washed their nets once every several months. A 34-year-old woman from the control district summarized what many described as their approach: “*I only wash it when it gets dirty. It’s not really about the time that has passed. It is about how the net looks.”* Respondents often mentioned that if a net was dirty, it needed to be washed to restore its aesthetic appeal before it could be used. Participants also stated that a dirty net could lead to contracting other diseases such as colds, lung disease, cough, and diarrhoea. A number of respondents mentioned that they washed their nets on a timetable similar to that of other laundry:

*You have to set yourself a day for laundry. I wash my clothes every Sunday because I am too busy with other chores during the week. So when I am washing clothes on Sunday, I wash my net as well.* (27 year old female, control district)

Few respondents mentioned barriers or negative attitudes about washing. However, several stated that the cost of soap made frequent washing prohibitive. As previously noted, a few reported that frequent washing caused holes to develop and made older nets more likely to tear. Most mentioned drying nets on a clothes line; several mentioned drying them in the shade, including hanging them on tree branches or inside the home near windows. However, drying demonstrations revealed potentially damaging practices such as hanging nets in direct sunlight or on tree branches, or spreading them out on the ground or on other rough surfaces outside such as fences.

#### Repair

Commonly cited reasons for repairing or replacing nets were to keep out mosquitoes, prevent malaria, and avoid the expense of treating malaria. Many participants stated that a mosquito could pass through a small hole and that it was important to repair even the smallest holes. A few participants noted that small holes became bigger and eventually unrepairable, rendering nets unusable for sleeping. Respondents often indicated that they would prefer purchasing a new net to repairing an old one; inability to afford a new net was often cited as the motivator for repair:

*[It] depends on one’s situation. If you have money, there is no need of sewing a net, you just buy a new one but if you are poor, you have to do it. So this is when you are poor.* (55 year old female, intervention district)

Respondents described and demonstrated several repair techniques. The most cited method was sewing holes shut with a needle and thread. Other methods included knotting, patching, and tying off with a rubber band, twine, or a similar piece of fabric. Equal numbers of respondents demonstrated sewing, knotting or tying their nets. Only a few demonstrated patching. A small number of respondents said they had never repaired a net and either did not know how, or would prefer buying a new net to repairing a torn one. None mentioned cost as a significant barrier to repair. Most agreed that purchasing a needle and thread were the only costs involved, and that both were readily available in local markets for 400–500 Ugandan shillings (about US$0.15-0.20). Most of those who mentioned patching said they could salvage material for patches at no cost from old clothes or worn nets.

Overall, respondents stated that they had the skills to repair nets. Most or all reported being able to sew or knot a hole closed, while about half said they could patch one. Most also demonstrated their ability to sew, tie, or knot a net with little difficulty. Participants were more divided on their preferred technique, with preference based on a combination of time involved, durability and net appearance. Estimates about the durability of sewing ranged from one day to permanent. Informants usually reported that sewing and patching were more durable. Knotting was said to consume too much netting fabric, shorten the net, and distort its shape, making it unattractive, too small to cover the bed, and likely to come untied quickly. Overall, informants described knotting as a ‘first aid’ measure that should be done as you look for a needle. Some respondents stated that a sewn net was ugly because threads might be left hanging or the net might look shabby if sewn with a different color thread. As one respondent explained:

*When you’re mending, you try to do something which remains smart … and uniform. [If it is not smart], you do not want other people to have access to it. Even the day when you are washing, you do not want to be seen.* (49 year old male, intervention district)

Some suggested that sewing would contribute to additional holes:

*… once you sew, the fabric near the repair is weakened in the process. So it doesn’t take you much longer to get another tear in the neighboring cloth… A future tear would come in another unrelated place*. (73 year old female, control district)

Negative opinions or barriers to patching were most often related to its time-consuming nature. Tying off holes with fabric, twine, or rubber bands was considered better than knotting because it did not consume as much netting fabric or distort the net’s shape and because it was less time consuming than sewing or patching.

#### Household roles

Women, wives or female heads of household were overwhelmingly reported as the household member responsible for net care and repair. Several respondents mentioned that older children were responsible for taking care of their own nets. In some instances, men reported they could undertake repairs and even demonstrated their sewing skills. Several explained that they were motivated to repair nets since they shouldered the financial burden when household members became sick with malaria and needed treatment. There was little mention of male involvement in the care of nets such as washing or daily maintenance.

#### Reported versus observed behaviour

Researchers noted discrepancies in some reported versus observed and demonstrated behaviour. A number of respondents reported having repaired nets that upon examination showed no signs of repair. A number reported that they had repaired nets by sewing or patching, that, upon inspection proved to have only knots with no indication of any longer term repair.

## Discussion

### Implications for programmes

To the authors’ knowledge, this is the first in-depth qualitative study to examine net care and repair attitudes and behaviour in Uganda. While the findings are specific to the two study districts, they illustrate some similarities and differences emerging from other recent studies elsewhere in Africa [18]. The findings are consistent with previous research showing that holes and damage to nets are common, and repair relatively uncommon [4–6, 9, 15, 18–21]. Respondent opinions on when nets are no longer useful for malaria prevention are consistent with studies in Senegal, Mali and Nigeria which have found that net users feel their nets are no longer worthy of repair when they are overly damaged or when holes are too large or too numerous [18–21]. Most users would prefer purchasing a new net to repairing an old one, but finances often limit their ability to do so [8, 18, 22–24].

This study confirms and expands upon previous findings about the strong social norms surrounding net hygiene and appearance [8, 18, 25]. Participants often mentioned cleanliness and aesthetics even before malaria prevention as a rationale for cleaning and repairing nets. Further, those who continued to use dirty or damaged nets were perceived as lazy or irresponsible, and participants expressed concern that others would perceive them in a similar way if they left nets dirty or unrepaired. In settings where many households have dirt floors, cook indoors with wood or charcoal, and use kerosene lamps, nets become dirty quickly. With the reported frequency of washing in the study area, LLINs are likely to reach their 20-wash lifetime limit within 12 to 18 months, potentially compromising fabric durability, insecticide effectiveness and overall longevity [13, 26]. Given the high value placed on hygiene, asking people to wash nets no more than four to five times per year may be unrealistic. Net distribution programmes should instead provide explicit instructions on proper washing technique and frequency to mitigate any potential confusion and encourage adherence to recommendations: *Use mild soap, stir and knead the net in a basin of water; avoid detergents and bleach, avoid scrubbing and beating on the rocks*. Programmes should also emphasize that nets are more delicate than clothing and thus require more careful washing. Net distribution and BCC campaigns that *demonstrate* as well as explain washing technique may be particularly useful [13, 27, 28]. The majority of nets observed hanging in study households were white, the colour of the campaign nets distributed in these two districts. Some pink and blue nets were observed and respondents explained they had purchased these nets from nearby markets. No data were collected about whether net colour influenced washing frequency. User-preference studies in Ghana, Kenya, Nepal, and Peru show that respondents often prefer coloured to white nets and sometimes stated explicitly that coloured nets show dirt less quickly [7, 28–30]. Thus net users with coloured nets may be willing to wait longer between washes [28]. The issue of whether net colour affects washing frequency should be explored in future research.

Differences between reported versus observed and demonstrated drying practices may highlight a general lack of awareness about drying as an integral part of the net care regimen and may also highlight confusion about whether nets should be dried in the sun or in the shade. Several studies have shown that washing and drying practices have varying effects on the bio-efficacy of Permanet and Olyset nets. There is some conflicting evidence about whether Olyset nets retain more insecticide when dried in the sun or in the shade [13, 31–34]. Until this issue is settled more conclusively, LLIN distribution programmes should continue to communicate the importance of drying nets out of direct sunlight and avoid possibly conflicting messages based on net brand. Programmes should also encourage people to dry their nets on a surface that will not produce snags or tears.

Participant responses regarding care reflect what BCC messages currently promote: that nets be stored or put out of the way during the day to prevent damage by children and rodents, the most commonly cited causes of damage. Messages aimed at preventing damage and keeping nets attractive to avoid the need for repair might resonate in contexts where repair is uncommon. BCC should continue to address the importance of daily tying up or, if nets are stored during the day, storing them in tightly sealed rodent- and pest-proof containers. BCC could also *demonstrate* as well as explain ways to hang nets to mitigate damage and perhaps provide guidance for households to identify safe places to dry nets. Involving children in net care responsibilities such as using caution around nets, making net care a part of daily chores, preventing younger siblings from playing with nets, and storing nets properly may help mitigate net damage. To counter perceptions of repaired nets as unattractive, BCC could position sewing and patching as more attractive than nets with unrepaired holes. Messages could also recast repair as an intelligent and efficient use of household resources rather than an admission of poverty. Messaging should emphasize that net repair is quick, easy, cheap and the sign of a caring and responsible person who is proactive about keeping his or her household safe and attractive.

Male involvement in repair and general maintenance of nets was mentioned more frequently by men than women. This is consistent with findings from another recent study that stated roles in care and repair were influenced by the gender of the respondent [18]. Nonetheless, males are an important audience for care and repair messages since men are often the primary decision-makers in Ugandan and many other African households. Messages that actively engage men may ensure that the entire household is both receptive to and involved in care and repair. A list of barriers and motivators mentioned by respondents and recommendations based upon those barriers and motivators – as well as additional recommendations from the research findings – can be found in Tables 3 and 4.

**Table 3:** **Barriers and facilitators to net care and repair (as mentioned by respondents)**

Behaviour	Motivators/facilitators	Barriers
Repairing a net	• Perceived risk of malaria due to holes	• Holes are too big or too many
	• Belief that mosquitoes can enter even a small hole	• Prefer to replace with new net if affordable
	• To save money on new nets or malaria treatment	• Duration (how long the repair would take; sewing or patching seen as slow)
	• Desire to be perceived as responsible and conscientious	• Potential unattractiveness of repair (distortion due to knotting, neatness of sewing, color of material and thread used)
	• Having a net that looks good (strong dislike of nets with holes)	• Not mentioned: Lack of materials, inability to sew, lack of knowledge of how to repair
	• Perception that repair can be fast and easy; especially knotting and tying off holes	
	• Awareness that small holes can get bigger	
	• Not having enough money to obtain a new net	
	• Realizing that a net that would have been considered unusable can still be used if repaired	
	• Needle, threat, and patching materials easily available at low to no cost	
	• How long the repair would last (sewing and patching seen as longer-lasting, knotting as quick to unravel)	
	• Men appear to approve of their wives caring for and repairing nets	
Washing a net	Motivators for NOT washing frequently:	Barriers to NOT washing frequently:
	• Frequent washing could cause holes	• Desire to be perceived as a clean and responsible person
	• Older nets more likely to tear during washing	• Frequent washing/cleanliness perceived as good care
	• Cost of soap (a few participants)	• Believing nets should be treated like clothes and washed following the laundry schedule
	• Understanding that frequent washing can reduce effectiveness of the ‘medicine’ in the net	• Belief that dirty nets could cause disease
		• Household factors like bedwetting and dirt floors
		• Confusion about proper washing instructions
		• Lack of proper washing instruction at distribution
Tying up a net or storing it when not in use	• Recognized as a good ‘routine’	• Tiring to do daily
	• Prevent damage to nets by children	• Easy to forget
	• Neat appearance	• Busy with morning rush to work/fields
	• Heads of households (men and women both) appear to approve in principle

**Table 4:** **Care and repair recommendations (based upon barriers and motivators identified by respondents and research findings)**

Care behaviour	Recommendations
Tying up a net or storing it when not in use	• Promote storing or tying up as an easy daily routine done by responsible, caring individuals that takes little to no time
	• Involve children and other household members
Washing nets	• Emphasize proper washing practices and frequency – wash net 3–4 times a year in a basin or bucket with water and mild soap, not detergent or bleach
	• Position nets as special, not to be treated like clothes, to be washed infrequently to protect the “medicine”
	• Keep nets tied up and/or stored when not in use to prevent dirt
	• Consider procuring coloured nets since they are less likely to show dirt
	• Manufacturers develop nets with insecticides that can stand a greater number of washes
	• Conduct trials of improved practices to explore how households can make washing less damaging and frequent
**Repair behaviour**	**Recommendations**
Repair (sewing, patching, knotting)	• Promote benefits of repair: malaria prevention purposes; saving money on purchasing a new net and on treatment for malaria
	• Emphasize the ease and short time required to repair small holes
	• Emphasize checking nets for holes routinely and repairing small holes immediately
	• Raise perceived dangers of delaying net repair: risk of malaria, financial costs of nets and treatment
	• Position repair as an intelligent and efficient use of resources
	• Position those who repair as responsible people who care for their family’s well-being and for having an attractive net and a well-kept home
	• Promote people who repair as people who are worthy of being appreciated and recognized
	• Create a norm of repairing by making it public (e.g., net repair as part of school homework)

### Strengths and limitations

Since these findings reflect the behaviour and attitudes of individuals in eastern Uganda, they can be used for LLIN BCC programmes in this area. The variety of methods used allowed researchers to gain an in-depth look at care and repair behaviour, attitudes, and social norms, and to help identify misconceptions or potentially damaging net care and repair practices. Nonetheless, this study is qualitative in nature and intended to represent the experiences of a relatively small number of respondents, so caution should be taken in extrapolating the findings to other contexts. Since demonstrations of washing, drying, care and repair demonstrations were simulated, participants may have left out certain details or been influenced by social desirability bias. Questions in the study guide focused primarily on repair and washing. While the guide also included questions on net handling and storage, these were less extensive. Differences in responses based on gender became apparent during the research, but guide questions were not specifically structured to examine gendered experiences in care and repair. As previously mentioned, both polyethylene and polyester nets were distributed during the mass campaign. The material and weave patterns are different, and it is possible that care and repair practices, particularly washing and drying, may impact LLIN integrity differently depending on net type. Nonetheless, this study was an examination of care and repair behaviour and attitudes, not net durability.

### Future research

Since researchers observed simulated washing and drying demonstrations rather than actual practices, more observational and perhaps even ethnographic studies would be useful to capture the full complexity of the behaviour and determine the extent of potential over-washing or other potentially damaging practices. Future research should work to identify care and repair strategies that are both effective and acceptable to net owners by recruiting a small number of net-owning households to test and critique different methods over short periods of time. This participatory research approach, known as trials of improved practices (TIPS), enables health programmes to quickly assess, refine and confirm the effectiveness of potential behaviour change interventions prior to large-scale implementation [28]. Such short-term trials offer promise for identifying less damaging washing and drying techniques and determining whether users are willing to wash nets less frequently. TIPS could also identify and build community consensus around acceptable and effective repair methods, the most feasible net storage strategies, and strategies for preventing and mitigating damage by children and other household stressors. A longitudinal study documenting whether coloured nets last longer and which, if any, colours last the longest could help determine the cost-effectiveness of procuring coloured nets. More research is also needed on gender roles surrounding care and repair and the types of gender-specific messages that may resonate most effectively.

## Conclusions

This study provides an in-depth look at barriers, motivators and attitudes surrounding net care and repair among select households in eastern Uganda. While findings are consistent with other studies regarding the causes and extent of net damage, this study offers some novel insights into the attitudes and social norms surrounding net care and repair in Uganda. Respondents considered aesthetics of their net and perceived the social stigma against dirty and damaged nets as especially important when deciding whether, when, and how to care for, repair and discontinue using their nets. BCC interventions may be able to capitalize on the social norm of owning an aesthetically pleasing net as a motivator to promote care and repair in other contexts. More in-depth, observational research is needed, particularly surrounding washing, drying, daily storage routines, and gender roles, in order to capture the full complexity of care and repair behaviour and help elucidate which behaviours require additional messaging versus refinement or clarification of existing messages and recommendations. Behaviour that prevents and mitigates net damage, such as care and repair, are important for maintaining net integrity and durability. Given the public health benefit and potential cost-savings of improving net integrity and thus durability, it is critical to understand the full range of barriers, motivators and attitudes surrounding net care and repair, to ensure that protective behaviour is promoted and that nets last their full, protective lifespan in the household.
